# Heart-Type Fatty Acid Binding Protein Is Associated with Proteinuria in Obesity

**DOI:** 10.1371/journal.pone.0045691

**Published:** 2012-09-18

**Authors:** Hui-Mei Chen, Chun-Xia Zheng, Qing Gao, Yong-Chun Ge, Zhi-Hong Liu

**Affiliations:** Research Institute of Nephrology, Jinling Hospital, Nanjing University School of Medicine, Nanjing, P. R. China; University of Tokushima, Japan

## Abstract

**Rationale:**

Lipid metabolism contributes to the formation of obesity-related glomerulopathy (ORG). Heart-type fatty acid binding protein (H-FABP or FABP3) is involved in lipid metabolism and was predicted to relate to renal lesions in obesity.

**Methods:**

A total of 28 patients with ORG were investigated, and renal tissue from 7 kidney donors served as controls. Db/db mice with albuminuria were treated with Simvastatin for 12 weeks.

**Results:**

Immunohistochemistry demonstrated the H-FABP staining in glomerular and tubular areas of patients with ORG, and the percentage of H-FABP in the glomerular area was significantly higher than in controls (15.8±1.62 versus 4.51±0.56%, *P*<0.001). Moreover, H-FABP expression correlated with proteinuria, high-density lipoprotein (HDL) cholesterol, waist circumference and the homeostatic model assessment – insulin resistance (HOMA-IR) among patients with ORG. Enhanced expression of H-FABP was also detected in the db/db mice, and expression increased from 8 to 20 weeks of age and was weakly related to increased albuminuria (*r* = 0.433; *P* = 0.020). Furthermore, H-FABP was co-localized with synaptopodin and demonstrated a podocyte pattern distribution. After Simvastation treatment, the urine albumin levels decreased with lipid levels and H-FABP expression in the glomeruli. The expression of H-FABP was related to Simvastatin treatment, albuminuria and triglycerides, while it was only linked with triglycerides and albuminuria (*r* = 0.643, *P* = 0.036).

**Conclusions:**

This study confirmed an association of H-FABP with the pathogenesis of clinical and experimental ORG, and suggests that such a process might be related to podocytes and lipid dysmetabolism.

## Introduction

Obesity is a major health problem, and its incidence is increasing worldwide [1]. It has been reported to contribute to the development of type 2 diabetes, hypertension, cardiovascular disease and chronic kidney disease (CKD) [2], [3]. This type of kidney disease is the so-called ‘obesity-related glomerulopathy (ORG)’ and is characterized by glomerulomegaly with or without focal segmental glomerulosclerosis (FSGS) [4]. Consequently, the incidence of ORG has increased over the last decade, and the epidemic of obesity has led to a progressive increase in number of cases of ORG in China [5]. Thus, understanding the pathogenesis is crucial to develop new therapies for its prevention and treatment.

Evidence shows that altered lipid metabolism, such as hyperlipidemia and increased free fatty acids (FAs), is an important characteristic of obesity and contributes to renal lesions [6]. Intracellular fatty acid-binding proteins (FABPs) are members of a multigene family encoding ∼15-kDa proteins, which allow the fatty acid to enter or exit the cellular cavity and thereby assist with the cell injury and death induced by the FAs [7]. Interestingly, we previously demonstrated that lipid dysmetabolism in involved in the development of ORG, and heart-type fatty acid binding protein (H-FABP or FABP3) is especially up-regulated in the glomeruli [8].

H-FABP is a member of the FABP family and mainly expressed in the heart and skeletal muscle [9]. Kimura et al. [10] first described that H-FABP is present in human glomeruli and is localized largely along the capillary wall. As an intracellular fatty acid-binding protein, however, the target cells in the glomeruli and their potential function in renal disease remain unclear. We recently continued our study of H-FABP, and primary data show that H-FABP in the glomeruli is co-localized with podocytes. Podocyte lesions have been proven to play a critical role in the development of many glomerular diseases, including ORG [11], [12].

Thus, regarding the function of H-FABP in lipid metabolism and its relationship to podocyte lesions, we postulated that up-regulation of H-FABP in the glomeruli of obese patients might contribute to podocyte dysfunction and promote the development of ORG. In this study, we aimed to elucidate the factors of lipid metabolism that related with H-FABP expression and whether the changes of H-FABP are involved in podocyte lesions and renal damage. For these purposes, we used humans with ORG and obese db/db mice to investigate the following: 1) the local distribution of H-FABP expression in glomeruli in cases of clinical and experimental ORG; 2) the association between H-FABP expression, lipid variables, podocyte lesions and renal damage in humans and mice; and 3) the effect of anti-lipid drug treatment on lipid metabolism and the responding H-FABP expression and renal damage in the db/db model.

## Methods

### Human subjects

Twenty-eight Chinese patients with obesity, proteinuria, and biopsy-proven obesity-related glomerulopathy (ORG) were enrolled from a single unit (Research Institute of Nephrology, Nanjing University School of Medicine, PR China). Biopsies in this institute were performed by the same group of clinicians, and renal biopsy specimens were examined by experienced nephrologists after preparation. Analysis included light microscopy, immunohistology, and electron microscopy. A final diagnosis was made for each patient on the basis of both clinical and histological investigations.

The diagnosis of ORG was established by [3], [5]: (1) obesity (BMI of 28 kg/m^2^ or greater), (2) positive proteinuria (urinary protein excretion of 0.4 g/24 h or greater), and (3) presentation with obesity-associated FSGS with glomerulomegaly or obesity-associated glomerulomegaly alone. Idiopathic cases of FSGS and minimal change disease were carefully ruled out according to clinical and histological characteristics, including variants in glomerular size, diffuse foot process effacement, and segmental glomerular scarring. Other underlying conditions that could cause FSGS or glomerulomegaly were excluded carefully, such as diabetic nephropathy and hypertensive nephrosclerosis.

Seven non-obese kidney donors were used as controls. Donors were matched for sex and age and had no history of hypertension, obesity, diabetes, or renal diseases. Written informed consent was obtained from all subjects prior to participation in the experimental protocol. The study was approved by the institutional review board of Nanjing University School of Medicine, and consistent with the Declaration of Helsinki.

Patient charts were reviewed for age, sex, and presenting clinical and laboratory data at the time of renal biopsy. Physical examinations, routine biochemical determinations, and 24-hour urine samples, which were used to measure daily proteinuria, were obtained. Plasma insulin concentrations were measured by means of radioimmunoassay (supplied by the Research Institute of Diabetes Mellitus, Chengdu, PR China). Insulin resistance was quantified using the Homeostasis Model Assessment of Insulin Resistance (HOMA-IR), a model used successfully for evaluating insulin sensitivity in large studies [13].

### Animals and animal care

C57BL/Ksj db/m normal and db/db diabetic mice were purchased from Jackson Research Laboratory (USA) and housed in our animal center. Mice aged 8 weeks were randomly divided into three groups as follows: A) db/m mice receiving saline (normal control); B) db/db mice receiving saline (diabetic control); C) db/db mice receiving Simvastatin (40 mg/kg/day p.o.). Simvastatin was purchased from Merck, Sharp & Dohme Co (Hangzhou, China). During the experiment, animals were given *ab libtum* access to food and housed in the laminar flow cabinet with a 12 h/12 h dark/light cycle. After 4, 8 and 12 weeks of treatment, 6 mice per group (the male to female ratio was 1∶1) were randomly chosen for body weight measurement and urine sample collection. The mice were then sacrificed and blood and kidney samples were collected. Animal experiments were approved by the Nanjing University School of Medicine Animal Ethics Committee.

Blood glucose levels were measured using an automated blood glucose reader (Accu-Chek, Roche). Urinary albumin and creatinine were determined using mouse-specific ELISA (Albuwell M kit) and Creatinine Companion kits (Exocell). Mouse serum creatinine, cholesterol, triglycerides, high-density lipoprotein (HDL) cholesterol and low-density lipoprotein (LDL) cholesterol were measured by an automated chemistry analyzer (Aeroset, Abbott, USA) using commercial kits (Abbott).

### Light microscopy

The human and mouse kidneys were fixed in 10% formaldehyde, embedded in paraffin, cut into 2 µm sections and stained with Periodic acid-Schiff (PAS). The pathological changes were observed under a light microscope. Photographs were obtained and quantitatively analyzed for morphology with SPI analysis software. For the human samples, roughly 50 glomeruli from a single needle biopsy were randomly selected and the percentages of global or segmental sclerosis were evaluated. For db/db mice, glomerular (G) and Bowman's capsule (B) areas were carefully traced by hand. G areas and B areas were measured using a digitizer KS-400 Imaging System. The ratio of G/B volume was calculated by the following formula: (G area/B area)^3/2^ [14].

### Immunohistochemistry

For H-FABP immunohistochemistry staining, the renal tissues were embedded in paraffin and fixed by transcardiac perfusion with PBS containing 4% paraformaldehyde. The slides were incubated with primary antibodies of H-FABP (ab28723 for human samples & ab16916 for mouse models, Abcam, Cambridge, MA) at room temperature for 1 h. Envision immunohistochemical staining was used and sections were developed with DAB after 30 minutes, followed by counterstaining with hematoxylin. The slides were observed under a light microscope. The H-FABP-positive area was quantitatively determined with Image Pro Plus 6.0 software.

For H-FABP immunofluorescence staining, frozen sections were incubated with the primary antibodies anti-H-FABP antibody (ab28723 for human and ab16916 for mice, Abcam, Cambridge, MA) and anti-synaptopodin antibody (Fitzgerald, Concord, CA), which was followed by CY3-conjugated or fluorescein isothiocyanate (FITC)-conjugated secondary antibodies. Immunofluorescence microscopy was performed using confocal microscopy (LSM 510; Carl Zeiss, Jena, Germany).

Additionally, immunohistochemical staining was performed with fibronectin-specific polyclonal anti-mouse antibody (Santa Cruz Biotechnology, Santa Cruz, CA). For evaluating the fibronectin score in db/db mice, the percentages of area stained for fibronectin were graded as follows: 0, staining absent to 5%; 1, 5 to 25%; 2, 25 to 50%; 3, 50 to 75%; and 4, >75%. A total of 20 randomly chosen glomeruli per mouse were graded and an investigator who was masked to sample identity performed the image analysis [15].

### Immunoelectron microscopy

Renal tissues were fixed by transcardiac perfusion with PBS containing 4% paraformaldehyde, dehydrated and embedded in LR white (Electron Microscopy Sciences). Ultrathin kidney cortical sections (70 nm) were mounted onto Formvar/carbon-coated nickel grids (Electron Microscopy Sciences). Aldehyde quenching with 0.05 mol/l glycine and antigen retrieval with citrate buffer (95°C for 10 minutes) were performed. After blocking, the tissues were incubated with rabbit anti-H-FABP antibody overnight at 4°C, followed by a donkey anti-rabbit antibody conjugated to 10 nmol/l gold particles. After rinsing, grids were fixed in 2.5% glutaraldehyde in 0.1 mol/l phosphate buffer and post-stained with uranyl acetate and lead citrate. The location of H-FABP was observed under an electron microscope.

### Statistical analysis

Data were analyzed using SPSS version 13.0 (SPSS Inc., Chicago, IL). Comparisons between groups were performed using Student's t-test. Relationships between parameters were analyzed using a Pearson or Spearman correlation coefficient. Multivariate analysis for related variables was performed using stepwise linear regression. Two-tailed *P* values less than 0.05 were considered statistically significant.

## Results

### 1. Increased H-FABP expression in the glomeruli of patients with ORG

**Figure 1:**
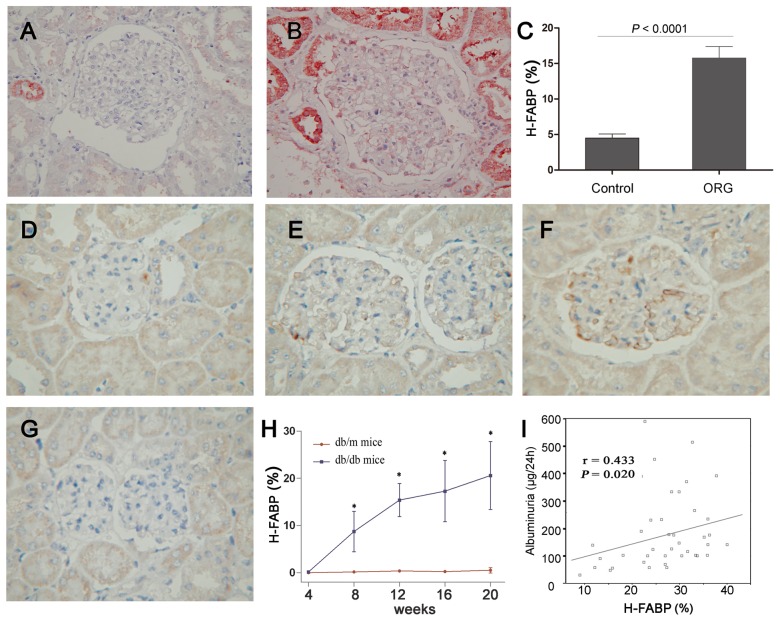
H-FABP in the glomeruli of patients with ORG and in db/db mice. **A–C:** H-FABP immunostaining in normal (A) and ORG-affected (B) glomerulus. It was shown that the expression of H-FABP was significantly higher in patients with ORG than in controls (C, *P*<0.0001). **D–H:** Mouse H-FABP expression from 8 week db/db (D), 12 week db/db (E), 20 week db/db (F) and 20 week db/m control (G) mice. The percentage of the H-FABP-positive area in the glomeruli increased with age in db/db mice and was significantly higher than in db/m mice (H). **I:** Correlation of H-FABP expression with the albuminuria level, Pearson's correlation test. Data are presented as the mean ± SD.

Immunostaining of renal sections from patients with ORG and from healthy controls showed strong H-FABP expression in human ORG lesions. The glomeruli of the healthy kidneys contained only a few H-FABP-positive areas (Figure 1A). In contrast, the glomeruli of patients with ORG showed obvious expression of H-FABP, and the deposits of H-FABP along the capillary walls were observed clearly (Figure 1B). The mean percentage of positive H-FABP expression in the glomeruli of patients with ORG was significantly higher than that of healthy controls (15.8±1.62 versus 4.51±0.56%, *P*<0.0001, Figure 1C). In addition, the strong positive areas of H-FABP were found in the tubular areas of both healthy controls and ORG patients. These observations suggest a possible relationship of H-FABP expression in glomeruli with ORG, and this relationship was further explored with regards to the progression of ORG.

As lipid dysmetabolism contributes to proteinuria in obesity, we hypothesized that patients with ORG have greater proteinuria and higher lipid levels, which would elevate expression of H-FABP in the glomeruli. To test this hypothesis, 28 patients with ORG were screened for H-FABP expression, renal damage and metabolic conditions (Table 1). Pearson correlation analysis showed that the positive area of H-FABP in the glomeruli correlated univariately with proteinuria levels (*r* = 0.537, *P* = 0.030). In addition, it was directly associated with waist circumference (*r* = 0.633, *P* = 0.020) and HOMA-IR (*r* = 0.725, *P* = 0.003), but inversely related to HDL cholesterol levels (*r* = −0.601, *P* = 0.001). These findings agreed with the elevated expression of H-FABP in patients with ORG and its significant correlations with renal damage or dysmetabolic factors.

**Table 1:** The correlation of H-FABP expression with renal damage and metabolic disturbance in patients with obesity-related glomerulopathy.

	H-FABP-positive area in glomeruli (%)	
	Correlation	*P*
Proteinuria	**0.537**	**0.030**
Creatinine clearance	0.304	0.192
Mean % sclerosis	0.083	0.674
Mean % global sclerosis	0.121	0.540
Mean % segmental sclerosis	−0.100	0.613
Age	0.058	0.768
Body mass index	0.141	0.475
Waist circumference	**0.633**	**0.020**
Waist-hip ratio	0.475	0.101
Mean blood pressure	−0.003	0.989
Total cholesterol	0.052	0.792
Triglycerides	0.077	0.687
High-density lipoprotein cholesterol	**−0.601**	**0.001**
Low-density lipoprotein cholesterol	−0.098	0.621
Glucose	0.213	0.296
Insulin	0.436	0.071
HOMA-IR	**0.725**	**0.003**
Uric acid	−0.286	0.140

Abbreviations: HOMA-IR, Homeostasis Model Assessment of Insulin Resistance.

At the same time, a group of patients with idiopathic FSGS were analyzed. Similar to the patients with ORG, those with FSGS presented with increased proteinuria and lipid dysmetabolism compared with healthy controls (Table S1). The positive percentage of H-FABP in the glomeruli was higher in patients with ORG and FSGS than in controls (Figure S1). In detail, the levels of proteinuria and lipid parameters were significantly higher in patients with FSGS than in patients with ORG. In contrast, the expression of H-FABP appeared to be lower in patients with FSGS compared to patients with ORG (11.9±2.08 versus 15.8±1.62%, *P* = 0.060, Figure S1).

### 2. H-FABP expression in the glomeruli of db/db mice

Experimental db/db mice were the model for obesity/diabetes and its related renal damage (Table 2). By 8 weeks of age, all db/db mice became obese and experienced a 40% increase in body weight compared to the db/m control mice. The db/db mice had higher levels of glucose, cholesterol, triglycerides, HDL and LDL, and a progressive increase in these factors was seen between 8 weeks and 20 weeks of age. Compared to the db/m mice, the db/db mice had higher urinary album from 8 weeks of age.

**Table 2:** Characteristics of db/db mice in renal damage and dysmetabolism.

	Body mass(g)	Glucose(mmol/L)	Cholesterol(mmol/L)	Triglycerides(mmol/L)	Creatinine(mmol/L)	Albuminuria (µg/24 h)	HDL Cholesterol(mmol/L)	LDL Cholesterol(mmol/L)
**db/m 8 weeks**	19.5±1.80	8.20±2.40	1.62±0.48	0.82±0.22	26.0±5.00	22.8±2.18	0.76±0.10	0.11±0.04
**db/db 8 weeks**	27.7±0.59 *	25.0±2.17**	2.66±0.20*	1.85±0.30*	27.8±3.23	86.9±19.8**	1.91±0.17*	0.12±0.06
**db/m 12 weeks**	22.0±0.90	8.60±2.50	1.12±0.38	0.92±0.28	29.0±4.50	23.5±3.08	0.86±0.04	0.12±0.06
**db/db 12 weeks**	38.7±0.55*	34.3±2.67**	3.16±0.57*	1.91±0.39*	31.3±4.70	168±34.2**	1.87±0.17*	0.18±0.02*
**db/m 16 weeks**	28.2±0.30	8.80±2.60	1.48±0.32	0.78±0.34	30.0±2.80	24.8±4.17	0.56±0.07	0.10±0.04
**db/db 16 weeks**	45.4±1.44**	42.2±4.05**	3.41±0.55*	2.22±0.38*	40.2±4.08*	202±44.8*	1.33±0.15*	0.17±0.06*
**db/m 20 weeks**	29.3±0.95	9.17±1.42	1.81±0.34	1.06±0.22	29.4±2.42	29.5±2.03	0.71±0.09	0.11±0.06
**db/db 20 weeks**	48.8±1.16**	43.8±4.23**	3.22±0.56*	2.43±0.37*	47.8±4.78**	244±62.0**	1.69±0.17*	0.19±0.04*

Note: Data expressed as the mean ± SD or count. *P<0.05, ** *P*<0.01 versus db/m mice with the same age.

Abbreviation: HDL: High-density lipoprotein cholesterol; LDL: Low-density lipoprotein cholesterol.

Enhanced expression of H-FABP was detected in the db/db mice from the 8^th^ week onwards to the 12^th^ and 20^th^ weeks (Figure 1D–F), while it was almost undetectable in the db/m mouse kidneys at 20 weeks (Figure 1G). The densities of H-FABP increased with age from 9.54 % at 8 weeks to 20.6 % at 20 weeks (Figure 1H). Moreover, examination of the db/db mice demonstrated weak correlations between H-FABP expression and albuminuria (*r* = 0.433; *P* = 0.020, Figure 1I). This conclusion agreed with the finding of increased expression of H-FABP and its correlation with renal damage in human patients with ORG.

### 3. Co-location of H-FABP and maker for podocytes

Consistent with published data [10], we have described that H-FABP is distributed along the capillary walls, where the podocytes have been proven to be present [16]. Regarding the multiple functions and the critical roles of the podocytes, we used synaptopodin, an actin-associated protein presenting in podocytes, to test the localization of H-FABP.

**Figure 2:**
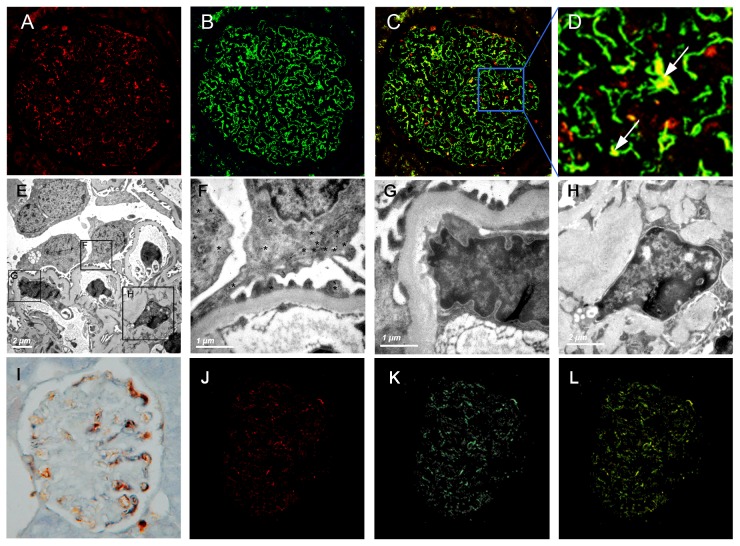
Characterization of H-FABP expression in patients with ORG and in db/db mice. **A–D:** ORG-affected kidney sections were examined for expression of H-FABP by immunofluorescence and confocal analysis: (A) H-FABP in red; (B) synaptopodin in green; (C) merge with original magnification ×400 and (D) merge with original magnification ×1640. It was clearly shown that H-FABP was co-localized with synaptopodin in cases of ORG, which is pointed to with white arrows. **E–H:** Immunoelectronic analysis was conducted to test the pattern of H-FABP expression, and staining was detected by H-FABP (E, *, ×8,000) in the foot process and the cytoplasm of podocytes (F, ×∼40,000), but not in the endothelial cell (G, ×∼40,000) or the mesangial areas (H, ×∼20,000). **I:** mouse H-FABP expression was examined by double immunohistological analysis in 20 week db/db mice, presenting H-FABP (in yellow), synaptopodin (in blue) and co-located (in black) staining. **J–L:** mouse H-FABP expression was examined by double immunofluorescence and confocal analysis in 20 week db/db mice: (J) H-FABP in red; (K) synaptopodin in green; and (L) merge showing co-localization in yellow.

It was shown that the H-FABP staining (Figure 2A) presented a similar distribution pattern with synaptopodin, in a “podocyte-like pattern” along glomerular capillary walls (Figure 2B). The two proteins almost overlapped under confocal microscopy and showed the yellow dots in Figure 2C & D. Immunoelectronic microscopy confirmed the scattered distribution of H-FABP in the foot process and the cytoplasm of the podocytes (Figure 2 E & F). On the contrary, the dot of H-FABP could not be detected in the endothelial cells or in the mesangial areas (Figure 2 G & H).

For db/db mice, the increased H-FABP protein was also distributed in a “podocyte-like pattern” in the hypertrophied glomeruli, where was similar as the distribution of synaptopodin. The immunohistological staining showed the co-localization of synaptopodin and H-FABP, which were detected in black among db/db mice (Figure 2I). The double immunofluorescence staining and confocal microscopy confirmed the co-localization, and H-FABP and synaptopodin were almost overlapped in db/db mice, presenting yellow dots (Figure 2J–L).

### 4. Amelioration of renal changes together with H-FABP expression after anti-lipid therapy

**Figure 3:**
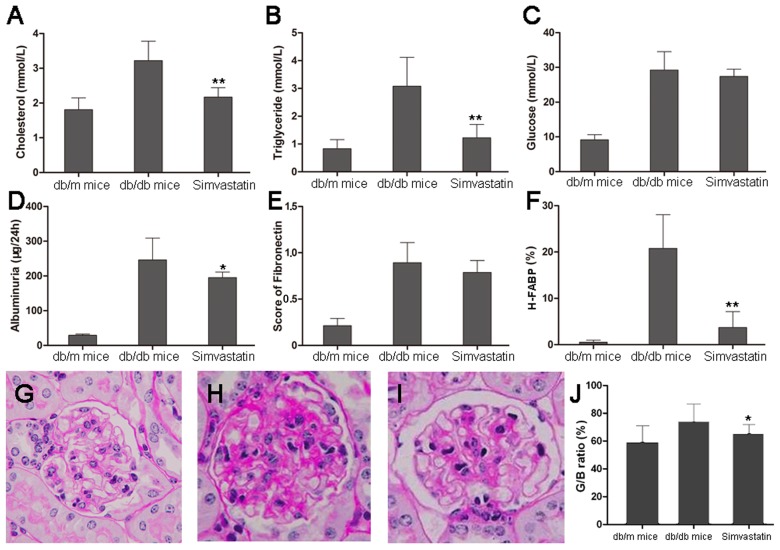
Simvastatin treatment in db/db mice. **A–B:** Dyslipid metabolism was significantly improved after treatment with Simvastatin in (A) serum cholesterol and (B) triglycerides. **C:** The levels of glucose were not changed. **D:** The levels of albuminuria were also remarked decreased in db/db mice. **E:** The fibronectin score on histology did not show a significant difference between db/db mice with and without Simvastatin treatment. **F:** The expression of H-FABP was significantly decreased in db/db mice with Simvastatin treatment compared to db/db mice without Simvastatin treatment. **G–J:** histological changes were shown with Simvastatin treatment in (G) db/m mice, (H) db/db mice without Simvastatin treatment, (I) db/db mice with Simvastatin treatment and (J) quantitative changes in G*/*B ratio (ratio of glomerular and Bowman's capsule volume) showed a remarked decrement in db/db mice with Simvastatin treatment. Data are presented as the mean ± SD. * *P*<0.05; ** *P*<0.01 versus db/db mice without Simvastatin treatment.

By light microscopy, the appearance of db/db mice is very similar to ORG, showing glomerular hypertrophy and mesangial matrix expansion. Selected images from db/m and db/db male mice were presented in Figure 3. In db/m mice, the outer cortical glomerulus was of normal size and configuration (Figure 3G). In distinction, the most severely affected glomerulus from a db/db mouse kidney shown appeared dramatically different (Figure 3H). The visceral epithelial cells were swollen and appeared prominent. The glomerular capillary basement membranes appeared thickened, and the peripheral capillary loop appeared collapsed. The mesangium was diffusely and markedly expanded with matrix material.

As pharmacological improvement of dyslipidemia with Simvastatin could ameliorate progression of obesity/diabetic nephropathy, we used the Simvastatin-treated mouse model to test its effect on H-FABP expression in cases of ORG. The levels of cholesterol and triglycerides decreased sharply after 12 weeks of Simvastatin treatment (*P*<0.001; Figure 3A & B). No profound changes in blood glucose levels were observed (*P* = 0.261, Figure 3C). After 12 weeks of treatment, the urine albumin levels were markedly decreased (*P* = 0.012, Figure 3D).

The number of cells in the glomeruli decreased and the widened mesangial region was alleviated by Simvastatin, as shown by histology (Figure 3G–I). The ratio of glomerular and Bowman's capsule volume (G/B) was significantly decreased in db/db mice with Simvastatin treatment compared to db/db mice without Simvastatin treatment (*P* = 0.028, Figure 3J). However, the fibronectin score was similar between the Simvastatin-treated group and the db/db control group (*P* = 0.164, Figure 3E).

The expression of H-FABP in the glomeruli was dramatically reduced after Simvastatin treatment compared with db/db control mice (*P*<0.001, Figure 3F). Using univariate linear regression analysis, the expression of H-FABP was shown to be significantly associated with Simvastatin treatment, albuminuria, and triglycerides but not with glucose and other lipid parameters (Table 3). Multiple regression analysis showed that H-FABP expression was only associated with triglycerides and albuminuria (*r* = 0.643, *P* = 0.036).

**Table 3:** The correlation of H-FABP expression with renal damage and metabolic disturbance in db/db mice.

	H-FABP-positive area in glomeruli (%)	
	Correlation	*P*
Albuminuria	**0.433**	**0.020**
Total cholesterol	0.141	0.237
Triglycerides	**0.589**	**0.005**
High-density lipoprotein cholesterol	0.208	0.397
Low-density lipoprotein cholesterol	0.076	0.648
Glucose	0.045	0.792
Insulin	0.346	0.090
Simvastatin treatment	**−0.687**	**<0.001**

## Discussion

It has been widely reported that lipid accumulation is related to renal damage [17]. As lipid-binding proteins, FABPs have proposed roles in fatty acid metabolism and been proven to be involved in the pathological events of the kidney [18], [19], [20]. We have long been interested in the mechanism of obesity-related glomerulopathy (ORG), which is an increasing epidemic syndrome of the kidney. Our previous study demonstrated that the epidemic of obesity had led to a progressive increase in the number of patients diagnosed with ORG during the last decades [5]. We further focused on H-FABP from gene expression profiles of the glomeruli, which was a protein in the FABP family, and showed an increased expression of H-FABP in ORG [8]. In this study, we confirmed the up-regulation of H-FABP in the glomeruli of ORG cases. We also showed that the over expression was mainly in the podocytes and might be involved in the progression of human ORG. The results of the db/db model were in agreement with the findings from human subjects. Moreover, the lipid proteins were accompanied by a reduction of H-FABP and renal lesions after the anti-lipid treatment in db/db mice.

ORG is a renal syndrome that is mainly characterized by increasing proteinuria followed by decreasing glomerular function [3], [21]. Renal biopsies showed that glomerulomegaly and FSGS are the most common histological lesions in patients with ORG. Experimental studies and clinical data show that obesity and its related metabolic disorders are important modifiable risk factors for this disease [22]. Dyslipidemia is one of the obvious characteristics of obesity, which might contribute to renal damage in patients with diabetes or obesity [23]. However, a correlation between lipid levels and renal impairment in patients with ORG could not be shown in our previous data [11]. It seemed that these serum markers for lipid metabolism, including cholesterol, triglycerides, high-density lipoprotein (HDL) cholesterol and low-density lipoprotein (LDL), might not be sensitive enough to reflect renal damage, although previous therapeutic interventions could not be ruled out.

Conversely, the expression of H-FABP in the glomeruli showed a tight association with the pathogenesis of ORG. We previously used a microdissection technique combined with Affymetrix microarray analysis to show that the expression levels of the LDL receptor, SREBP-1, and H-FABP all increased remarkably. These genes related to lipid dysmetabolism were one of the subgroups that changed significantly in the glomerular gene expression profiles of ORG [8]. In the present study, we confirmed with histological staining that the expression of H-FABP was significantly higher in the glomeruli of patients with ORG compared to controls (Figure 1A–C). Db/db mice further demonstrated stronger H-FABP expression in the glomeruli compared to db/m mice (Figure 1 D–G). It was the first time indicating that H-FABP was a possible marker linking with lipid metabolism and renal damage. But, our findings could not rule out the effect of other genes detected by microarray analysis. Further studies were continued to unveil the association of lipid dysmetabolism with ORG.

More interestingly, increased expression of H-FABP was directly related with proteinuria level in humans with ORG (Table 1). In db/db mice, the densities of H-FABP in glomeruli increased with age (Figure 1H) and was also associated with albuminuria (Figure 1I, *P* = 0.020). After Simvastatin treatment, the expression of H-FABP was reduced and independently correlated with anti-lipid treatment and albuminuria levels. Proteinuria and albuminuria are the hallmarks of obesity-related renal damage [24]. Thus, we proposed that the H-FABP expression in the glomeruli might be involved in the genesis of ORG and contribute to the development of proteinuria and the progression of renal damage.

Glomerular hypertrophy is one of the typical features in db/db mice, which may be due to alteration of glomerular hemodynamics. The G/B ratio is a marker for glomerulomegaly and elevated G/B ratio was observed under conditions with high glomerular filtration rate (GFR) [25]. Our results showed the G/B ratio was markedly reduced by Simvastatin treatment (Figure 3I). It suggested that Simvastatin might attenuate glomerular hypertrophy through ameliorating lipid dysmetabolism. In addition, the score of fibronectin was found to be dramatically increased in db/db mice, but it was similar between before and after Simvastatin treatment (Figure 3E). It indicated that Simvastatin played a weak role on chronic lesions. Therefore, H-FABP expression in the glomeruli might be involved in renal lesions, especially in glomerular hypertrophy.

However, it was difficult to clarify that the increased expression of H-FABP was specifically related with ORG as opposed to general renal damage. To clarify this issue, we chose another glomerular disease, idiopathic FSGS. It is a primary nephritis and similarly characterized by proteinuria, glomerulosclerosis and renal dysfunction [26]. Compared with healthy controls, both patients with ORG and FSGS showed higher H-FABP in their glomeruli (both *P*<0.001; Figure S1). If H-FABP of the glomeruli was postulated only to reflect grades of renal damage, there would be stronger expression of H-FABP in FSGS patients, as FSGS patients showed significantly higher levels of proteinuria (Table S1), compared with ORG patients. However, the densities of H-FABP were remarkedly lower in patients with FSGS than in those with ORG (Figure S1). Combined the association of H-FABP expression with lipid metabolic parameters, including triglycerides and HDL, we suggested that H-FABP expression in glomeruli was a possible marker, linked with lipid metabolism, podocyte function and renal lesions in obesity. But, it was difficult of draw a conclusion whether H-FABP played a protective or detrimental role during such process related with obesity. Additionally, we could not find a general relation between H-FABP and synaptopodin expression under univariate analysis (data not shown) and the factor of podocyte loss should also be taken into account. Podocyte loss might lead to an inadequate expression of H-FABP in glomerular area, which would partly account for decreased expression of H-FABP in FSGS.

Another interesting finding of the current study is that the H-FABP in the glomeruli was mainly expressed in the podocytes. All of the confocal immunofluorescence, double immunohistological staining and colloidal gold immuno-dot assay confirmed the co-localization of H-FABP and the marker for podocytes. This co-localization has been described in both human cases of ORG and in db/db mice (Figure 2). We have shown that podocyte lesions in patients with ORG precipitated the development of proteinuria and the decrease in renal function, as well as glomerulosclerosis [11]. The association between lipid metabolism and podocytes has also been widely studied [16]. Some possibilities accounted for these relationships: 1) podocyte lesions resulting from the increment of H-FABP expression; 2) H-FABP playing a protective effect on podocyte by attraction of FFA; or 3) their coexistence in the presence of lipid dysmetabolism. Our results could not determine the underlying mechanism. Mayrhofer et al. [27] showed that a disrupted fatty acid metabolism in concert with an impaired antioxidant defense mechanism in podocytes might play a role in the early stages of podocyte lesions. We postulated that the effect of H-FABP on proteinuria related with obesity might be mediated by the function of podocytes.

Additionally, our previous studies explored the relationship between glucose dysmetabolism and renal damage, showing insulin resistance (HOMA-IR) was a possible marker linked with protinuria of ORG patients [28]. The present study illustrated that H-FABP was univariately correlated with the level of HOMA-IR in ORG patients. However, no association of glucose level was detected with renal damage in ORG patients. Such no relation might be consequent to that: 1) glucose levels in patients varied mainly with difference diet and treatment in the retrospective studies; and 2) blood glucose levels were consistent in db/db mice without effective anti-glucose treatment. Moreover, there were few evidences about H-FABP roles in glucose metabolism. Considering the function of H-FABP in lipid metabolism, we stressed the association of H-FABP with lipid dysmetabolism and renal damage.

In conclusion, this study provided evidence from human ORG patients and a mouse model of obesity with renal damage that supporting the association of H-FABP with ORG pathogenesis. Direct correlation of H-FABP expression with proteinuria indicated that H-FABP could be a more sensitive marker than other lipid parameters. Although the mechanisms remain undiscovered, H-FABP expression in glomeruli is associated with lipid metabolism, which was involved in podocyte function and renal lesions in obesity. All of these processes may contribute to ORG development.

## Supporting Information

Figure S1 **The expression of H-FABP in the glomeruli of controls and in patients with FSGS and ORG.** It was shown that the expression of H-FABP was significantly higher in patients with ORG and FSGS than in controls (*P*<0.001). Although it seemed higher in patients with ORG compared to patients with FSGS, it did not reach statistical significance (*P* = 0.060).
(TIF)

Table S1 **Clinical characteristics of patients with idiopathic FSGS and patients with ORG, compared with healthy controls.**
(DOC)
