# Supplementary figures and images for: Heart-Type Fatty Acid Binding Protein Is Associated with Proteinuria in Obesity
Source: PLoS One. 2012 Sep 18;7(9):e45691. doi: 10.1371/journal.pone.0045691 (PMC3445507; doi:10.1371/journal.pone.0045691)

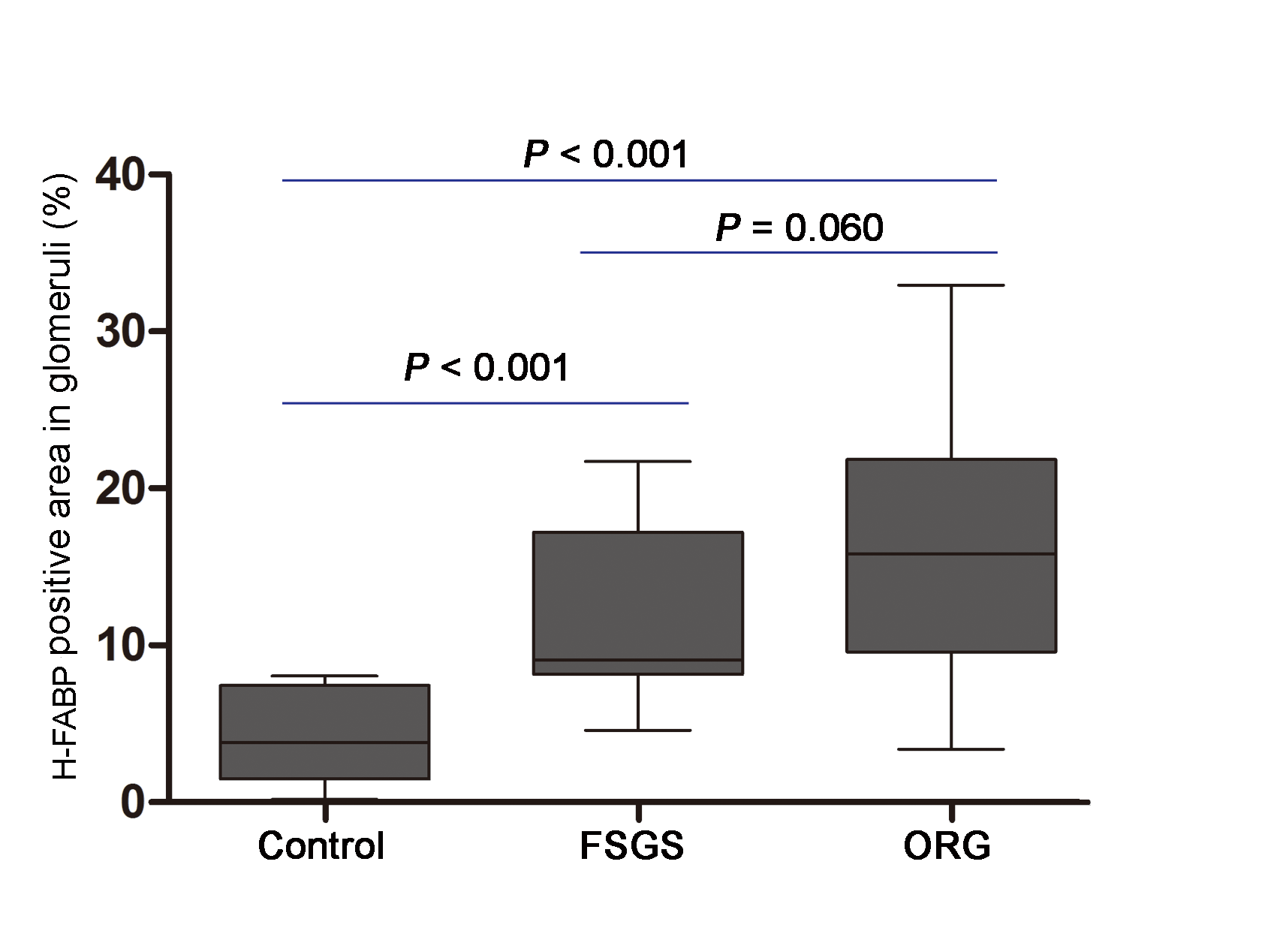

Supplement: Figure S1 — The expression of H-FABP in the glomeruli of controls and in patients with FSGS and ORG. It was shown that the expression of H-FABP was significantly higher in patients with ORG and FSGS than in controls (P<0.001). Although it seemed higher in patients with ORG compared to patients with FSGS, it did not reach statistical significance (P = 0.060). (TIF) [file pone.0045691.s001.tif]
