# Supplementary material for: Heart-Type Fatty Acid Binding Protein Is Associated with Proteinuria in Obesity
Source: PLoS One. 2012 Sep 18;7(9):e45691. doi: 10.1371/journal.pone.0045691 (PMC3445507; doi:10.1371/journal.pone.0045691)
Supplement: Table S1 — Clinical characteristics of patients with idiopathic FSGS and patients with ORG, compared with healthy controls. (DOC) [file pone.0045691.s002.doc]

**Table S1**: Clinical characteristics of patients with idiopathic FSGS and patients with ORG, compared with healthy controls.

|  | **Controls** | **Patients with FSGS** | **Patients with ORG** |
| --- | --- | --- | --- |
| N. of patients | 10 | 28 | 28 |
| Men/women | 6/4 | 18/10 | 18/10 |
| Age (y) | 35.3 ± 14.7 | 22.6 ± 6.32 ** | 36.9 ± 11.5 † |
| Body mass index | 23.6 ± 0.75 | — ^a^ | 31.7 ± 3.55 ** |
| Know duration of obesity (y) | — | — | 13.4 ± 5.88 |
| Mean blood pressure (mm Hg) | 91.0 ± 2.32 | 103 ± 16.1 | 103 ± 13.4* |
| Pulse blood pressure (mm Hg) | 47.1 ± 11.1 | 46.2 ± 5.09 | 48.9 ± 9.01 |
| Hypertension (%) | 0 (0%) | 12 (42.9%) ** | 11 (39.3%) ** |
| Known duration of proteinuria (mo) | — | 5.68 ± 4.37 | 37.4 ± 45.1‡ |
| Serum albumin (g/dL) | 4.27 ± 0.38 | 2.14 ± 0.64** | 4.09 ± 0.61‡ |
| Proteinuria (g/24 h) | 0.20 ± 0.10 | 9.44 ± 5.53** | 1.47 ± 1.36**‡ |
| Serum creatinine (mg/dL) | 0.60 ± 0.18 | 1.47 ± 0.88** | 0.85 ± 0.40‡ |
| Total cholesterol (mmol/L) | 3.68 ± 0.83 | 13.4 ± 3.09** | 5.36 ± 1.87‡ |
| Triglycerides (mmol/L) | 0.86 ± 0.22 | 5.05 ± 1.16** | 2.55 ± 1.28**‡ |
| HDL cholesterol (mmol/L) | 1.33 ± 0.37 | 2.03 ± 0.53** | 1.12 ± 0.26 ‡ |
| LDL cholesterol (mmol/L) | 2.27 ± 0.67 | 8.51 ± 2.13** | 3.35 ± 1.19 ‡ |

Note: Data expressed as the mean ± SD or count (percentage). ^a^: It was difficult to calculate accurate body mass index because of edema.

**P*<0.05, ** *P*<0.01 versus controls; † *P*<0.05, ‡*P*<0.01 versus patients with FSGS.

Abbreviation: FSGS: focal segmental and glomerulosclerosis; ORG: obesity related glomerulopathy; HDL: High-density lipoprotein cholesterol; LDL: Low-density lipoprotein cholesterol.
